# Treatment recommendation based on SYNTAX score 2020 derived from coronary computed tomography angiography and invasive coronary angiography

**DOI:** 10.1007/s10554-023-02884-0

**Published:** 2023-06-27

**Authors:** Shinichiro Masuda, Patrick W. Serruys, Shigetaka Kageyama, Nozomi Kotoku, Kai Ninomiya, Scot Garg, Alan Soo, Marie-Angele Morel, John D. Puskas, Jagat Narula, Ulrich Schneider, Torsten Doenst, Kaoru Tanaka, Johan de Mey, Mark La Meir, Antonio L. Bartorelli, Saima Mushtaq, Giulio Pompilio, Daniele Andreini, Yoshinobu Onuma

**Affiliations:** 1 Department of Cardiology, University of Galway, Galway, Ireland; 2 Department of Cardiology, Royal Blackburn Hospital, Blackburn, UK; 3 Department of Cardiothoracic Surgery, University Hospital Galway, Galway, Ireland; 4 Department of Cardiovascular Surgery, Mount Sinai Morningside, New York, USA; 5 The Charles Bronfman Institute for Personalized Medicine, Icahn School of Medicine at Mount Sinai, New York, USA; 6 Department of Cardiothoracic Surgery, Friedrich-Schiller-University of Jena, Jena University Hospital, Jena, Germany; 7 Department of Radiology, Vrije Universiteit Brussels, Brussels, Belgium; 8 Department of Cardiac Surgery, Universitair Ziekenhuis Brussel, Belgium, Belgium; 9 Division of Cardiology and Cardiac Imaging, IRCCS Ospedale Galeazzi Sant’Ambrogio, Milan, Italy; 10 Department of Biomedical and Clinical Sciences “Luigi Sacco”, University of Milan, Milan, Italy; 11 Department of Periooperative Cardiology and Cardiovascular Imaging, Centro Cardiologico Monzino, IRCCS, Milan, Italy; 12 Department of Cardiovascular Surgery, Centro Cardiologico Monzino IRCCS, Milan, Italy; 13 Department of Biomedical, Surgical and Dental Sciences, University of Milan, Milan, Italy

**Keywords:** Coronary artery bypass graft, Percutaneous coronary intervention, Coronary computed tomography angiography, Invasive coronary angiography, Treatment recommendation

## Abstract

The diagnostic performance of the SYNTAX score 2020 (SS-2020) when calculated using CCTA remains unknown. This study aimed to compare treatment recommendations based on the SS-2020 derived from coronary computed tomography angiography (CCTA) versus invasive coronary angiography (ICA). This interim analysis included 57 of the planned 114 patients with de-novo three-vessel disease, with or without left main coronary artery disease, enrolled in the ongoing FASTTRACK CABG trial. The anatomical SYNTAX scores derived from ICA or CCTA were evaluated by two separate teams of blinded core-lab analysts. Treatment recommendations were based on a maximal individual absolute risk difference in all-cause mortality between percutaneous coronary intervention (PCI) and coronary artery bypass graft (CABG) of 4.5% ([predicted PCI mortality] – [predicted CABG mortality]). The level of agreement was evaluated with Bland–Altman plots and Cohen’s Kappa. The mean age was 66.2 ± 9.2 years and 89.5% of patients were male. Mean anatomical SYNTAX scores derived from ICA and CCTA were 35.1 ± 11.5 and 35.6 ± 11.4 (p = 0.751), respectively. The Bland–Altman analysis showed mean differences of − 0.26 and − 0.93, with standard deviation of 3.69 and 5.23, for 5- and 10-year all-cause mortality, respectively. The concordance in recommended treatment for 5- and 10-year mortalities were 84.2% (48/57 patients) and 80.7% (46/57 patients), with Cohen’s κ coefficients of 0.672 and 0.551. There was moderate to substantial agreement between treatment recommendations based on the SS-2020 derived using CCTA and ICA, suggesting that CCTA could be used as an alternative to ICA when making decisions regarding the modality of revascularization.

**Supplementary Information:**

The online version contains supplementary material available at 10.1007/s10554-023-02884-0.

## Introduction

Coronary computed tomography angiography (CCTA) is a non-invasive diagnostic tool that is increasingly being used as an alternative to invasive coronary angiography (ICA) in patients with suspected coronary artery disease (CAD), which can provide important information on the extent and severity of CAD, as well as on the composition of atherosclerotic plaque and can identify plaque with high-risk features for adverse outcomes [1, 2]. The SYNergy between percutaneous coronary intervention with TAXus and cardiac surgery (SYNTAX) anatomical score is a scoring system based on ICA which assesses the anatomical extent and complexity of CAD and has become an established tool to help predict prognosis in patients undergoing percutaneous coronary intervention (PCI) or coronary artery bypass graft (CABG) [3, 4]. Through novel technology and improvements in image resolution assessment of the anatomical SYNTAX score using CCTA has proven to be accurate and comparable to evaluation using ICA [5]. The SYNTAX III REVOLUTION trial demonstrated that clinical decision-making between CABG and PCI based on CCTA, according to equipoise in four-year mortality as predicted by the SYNTAX score II, had high agreement with treatment decisions derived from ICA [6]. Of note, this randomized trial was virtual in nature since the concordance or discordance in decision-making between ICA and CCTA was unveiled to the operators (surgeon or interventional cardiologist) prior to the final definitive decision regarding percutaneous or surgical revascularization. In 2019, the SYNTAX score II was redeveloped using key angiographic and clinical variables available at the time of decision-making to create the SYNTAX score II 2020 (SS-2020), which now provides individual patients with a personalized predicted treatment benefit of CABG over PCI, in terms of their 5-year risk of having a major adverse cardiovascular/cerebrovascular event (MACE) and their 5- and 10-year risk of all-cause death [7]. The purpose of this study was to compare treatment recommendations based on the SS-2020 when derived using CCTA versus ICA in patients with de-novo three-vessel disease (3VD) with or without left main coronary artery disease (LMCAD), who were enrolled in the FASTTRACK CABG study, and had already been selected to have CABG by a conventional heart team that only had access to ICA. In other words, the study removed the virtual decision-making employed in the SYNTAX III REVOLUTION trial, and hence tested the real feasibility and safety of planning and performing a CABG based solely on the guidance of CCTA and fractional flow reserve derived from CCTA (FFR-CT).

## Methods

### Study population

This study was the interim analysis which included all 57 enrolled patients out of the planned 114 patients in the FASTTRACK CABG trial (NCT04142021) [8]. Briefly, the study is an ongoing, single-arm, multi-center, prospective, proof of concept study to assess the feasibility of planning and performing CABG based solely on CCTA and FFR-CT results in patients with de-novo 3VD with or without LMCAD. A ‘conventional heart team’ reviewed and assessed the ICA, however they were not involved in operational planning or surgical treatment, which was overseen by the “operating/CCTA heart team” that had sole access to CCTA and FFR-CT, without knowledge of ICA [8]. The flow-chart of the FASTTRACK CABG trial is shown in Supplementary Fig. 1. Clinical data were adjudicated by an independent clinical events committee. Written informed consent approved by the ethical committee of each site was obtained from all patients. The study complied with the declaration of Helsinki and good clinical practice.

### Image acquisition and analysis of CCTA

CCTA was performed exclusively at all four academic sites using the Revolution CT scanner (GE Healthcare, Milwaukee, WI, USA) which has a nominal spatial resolution of 230 microns along the X–Y planes, a rotational speed of 0.28 s, and a Z-plane coverage of 16 cm enabling imaging of the whole heart in one heartbeat [9, 10]. In select cases, a proprietary post-processing algorithm (SnapShot Freeze, GE Healthcare) was used for the additional correction of any residual motion artifacts. The protocol mandated the use of nitrates prior to CT acquisition and beta-blockers when heart rates were > 65 bpm.

### Anatomical SYNTAX score and SYNTAX score 2020 calculations

Anatomical SYNTAX score calculations derived from ICA and CCTA were performed blindly by two independent groups of experienced analysts of the academic core laboratory team. Coronary segments with a visual diameter stenosis > 50% in vessels ≥ 1.5 mm using CCTA or ICA were assessed and weighted according to their location in the coronary tree [11]. The SS-2020 was developed from the 10-year follow-up of the SYNTAXES trial and externally validated in four randomized trials (FREEDOM, BEST, PRECOMBAT, and EXCEL) and a large contemporary registry (CREDO-KYOTO cohort 2 and 3) of patients with 3VD, with or without LMCAD, treated with PCI or CABG [7, 12, 13]. The score, which uses two anatomical effect modifiers (the anatomical SYNTAX score and the presence of 3VD or LMCAD) and seven clinical variables (age, creatinine clearance, left ventricular ejection fraction [LVEF], chronic obstructive pulmonary disease, peripheral vascular disease, medically treated diabetes mellitus, and current smoking), predicts 5-year MACE defined as all-cause mortality, stroke, myocardial infarction, or repeat revascularization as well as 5- and 10-year all-cause mortality [7].

### Treatment recommendation based on SYNTAX score 2020

**Fig. 1:**
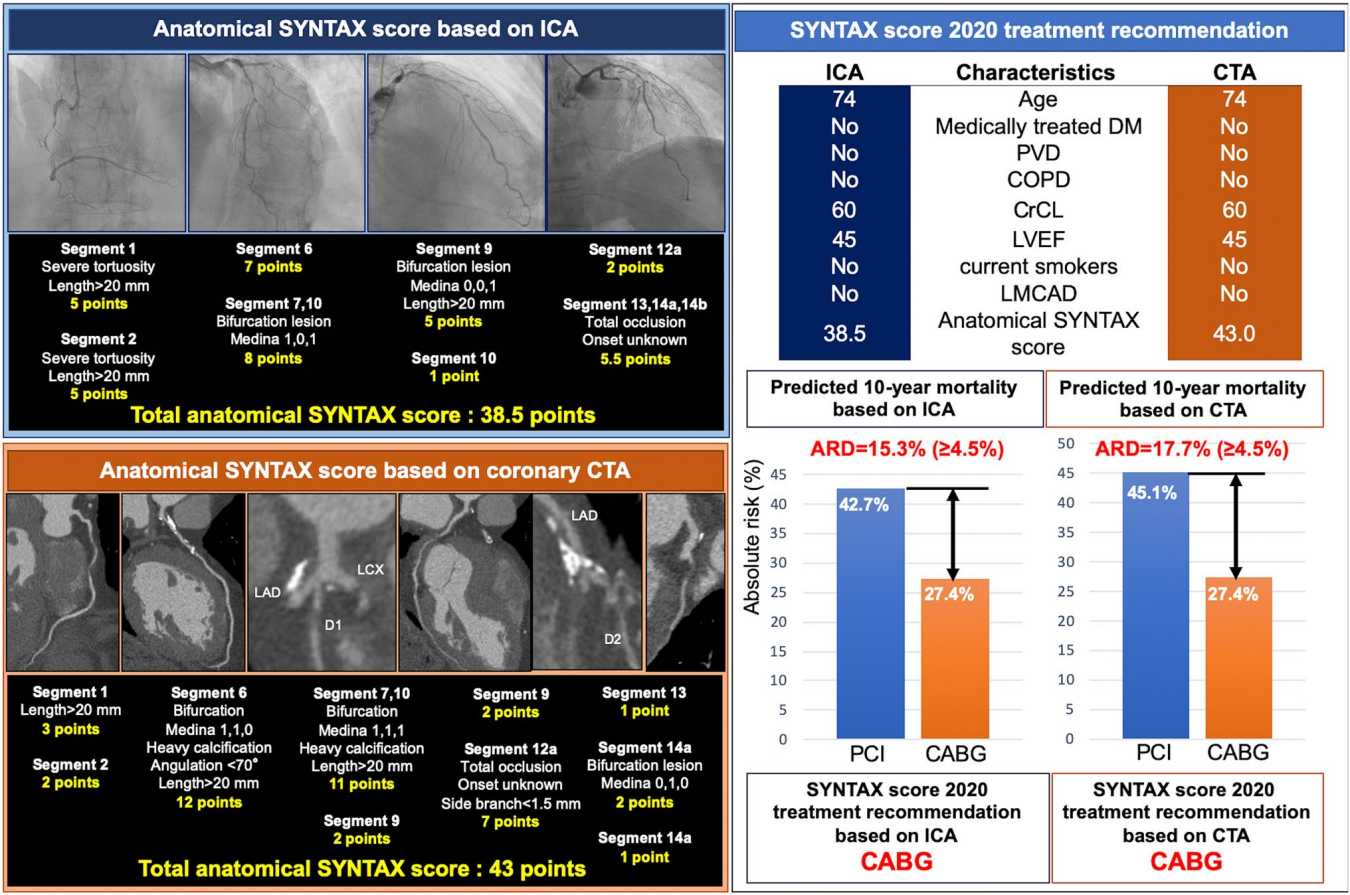
Representative case of SYNTAX score 2020 calculation. A 74-year-old man with creatinine clearance of 60 ml/min and LVEF of 45%. The anatomical SYNTAX score derived from ICA is 38.5, and from CCTA is 43.0. Predicted 10-year all-cause mortality based on ICA with PCI and CABG are estimated to be 42.7% and 27.4%, respectively. Alternatively, predicted 10-year mortality with PCI and CABG based on CCTA are estimated to be 45.1% and 27.4% respectively. Individual ARD was estimated to be 17.7% on ICA (42.7% [all-cause mortality with PCI] − 27.4% [all-cause mortality with CABG]) and 15.3% on CCTA (45.1–27.4%). Based on these findings, CABG is recommended by both ICA and CCTA. *ARD* absolute risk difference, *CABG* coronary artery bypass graft, *CrCL* creatinine clearance, *CCTA* coronary computed tomography angiography, *COPD* chronic obstructive pulmonary disease, *DM* diabetes mellitus, *ICA* invasive coronary angiography, *LMCAD* left main coronary artery disease, *LVEF* left ventricular ejection fraction, *PCI* percutaneous coronary intervention, *PVD* peripheral vascular disease

Treatment recommendation was made based on the individual absolute risk difference (ARD) between PCI and CABG for all-cause mortality at 5 and 10 years calculated by subtracting the predicted CABG mortality from the predicted PCI mortality. According to an external validation in a large contemporary registry, an individual predicted ARD in all-cause death at 5 years of < 4.5% and ≥ 4.5% offers a sensible cut-off for “equipoise of PCI and CABG” or “CABG better,” respectively [13]. A representative case of CCTA-based SYNTAX score calculation and treatment recommendation according to SS-2020 can be seen in Fig. 1.

### Statistical analysis

Continuous variables were expressed as mean ± standard deviation and were compared with the use of Paired t-test. Categorical variables were reported as numbers and percentages and were compared using the Chi-square test. Individual absolute risk differences (ARDs) between predicted PCI and CABG for mortality at 5- and 10-year were shown by scatterplot in descending order of magnitude according to the predicted ARD in mortality (survival benefit) for each patient. The dots in the scatter plots were connected with the use of locally estimated smoothing (LOESS) spline curves [13]. The Kappa value was used to assess the agreement of the treatment recommendation according to the SS-2020 derived by ICA and CCTA [14, 15], and the Pearson correlation coefficient was used to assess the correlations. The level of agreement between the two modalities was assessed by the Bland–Altman method, and Kohen’s Kappa [16]. A two-sided p-value < 0.05 was considered statistically significant. Analyses were performed with SPSS Statistics, version 27 (IBM Corp., Armonk, N.Y., USA).

## Results

### Baseline characteristics

Baseline characteristics are displayed in Table 1. The average age was 66.6 ± 9.1 with 89.5% of patients’ male. The mean body mass index (BMI) was 27.5 kg/m^2^, and 26.3% of patients had diabetes mellitus, but only one patient was insulin dependent. The mean LVEF was 55.9 ± 7.8%.

**Table 1:** Baseline patient characteristics

Characteristics	N = 57
Age, mean ± SD	66.6 ± 9.1
Male	89.5 (51/57)
Current smoking (%, n)	17.5 (10/57)
Diabetes mellitus (%, n)	26.3 (15/57)
On insulin use (%, n)	1.8 (1/57)
Hypertension (%, n)	89.5 (51/57)
Hyperlipidemia (%, n)	75.4 (43/57)
Family history of CAD (%, n)	29.8 (17/57)
Previous stroke (%, n)	3.5 (2/57)
Previous myocardial infarction (%, n)	10.5 (6/57)
Previous heart failure (%, n)	3.5 (2/57)
COPD (%, n)	8.8 (5/57)
PVD (%, n)	8.8 (5/57)
Creatinine clearance (ml/min) mean ± SD	79.9 ± 17.5
BMI (kg/m^2^), mean ± SD	27.5 ± 4.4
LVEF (%), mean ± SD	55.9 ± 7.8
Euro Score II, mean ± SD	1.05 ± 0.49

*BMI* body mass index, *CAD* coronary artery disease, *COPD* chronic obstructive pulmonary disease, *LVEF* left ventricular ejection fraction, *PVD* peripheral vascular disease, *SD* standard deviation

### Lesion characteristics and SS-2020 calculation

Lesion characteristics and the results of SS-2020 calculations between ICA and CCTA are shown in Table 2**.** The mean anatomical SYNTAX scores derived from ICA and CCTA were comparable (35.1 ± 11.5 and 35.6 ± 11.5, respectively P = 0.751), despite the total number of lesions per patient being significantly higher with CCTA (5.0 ± 1.5 vs. 6.0 ± 1.7, P < 0.001). The presence of a trifurcation lesion was significantly higher in ICA than CCTA (8.2% vs 1.7%, P < 0.005). The number of heavily calcified lesions was comparable among the two modalities (22.5% vs. 23.5%, P = 0.758), however the presence of severe tortuosity (7.1% vs. 0.0%, P < 0.001) and the frequency of lesions > 20 mm long (37.3% vs. 24.0%, P < 0.001) were both significantly higher with ICA than CCTA. Despite the above-mentioned individual differences, the global average anatomical SYNTAX scores were similar. Furthermore, the incorporation of clinical characteristics to construct the SS-2020 confirmed the comparability of the SS-2020 and the predicted risk of MACE/all-cause mortality following surgical or percutaneous treatment.

**Table 2:** Comparison of lesion characteristics and SYNTAX score 2020 calculations between two modalities

Characteristics	Assessment based on ICA	Assessment based on CCTA	P-value
Anatomical SYNTAX score, per patient	35.1 ± 11.5	35.6 ± 11.5	0.751
Lesion numbers, per patient	5.0 ± 1.5	6.0 ± 1.7	< 0.001
Left main disease	5.4 (15/280)	2.4 (9/280)	0.074
Components anatomical SYNTAX score			
Total occlusion	12.9% (36/280)	12.8% (44/344)	0.652
Trifurcation	8.2% (23/280)	1.7% (6/344)	< 0.001
Bifurcation	34.3% (96/280)	27.0% (93/344)	0.050
Medina 1,0,0	4.6% (13/280)	2.3% (8/344)	
Medina 0,1,0	5.0% (14/280)	4.7% (16/344)	
Medina 1,1,0	4.3% (12/280)	5.2% (18/344)	
Medina 1,1,1	7.1% (20/280)	3.8% (13/344)	
Medina 0,0,1	6.8% (19/280)	5.5% (19/344)	
Medina 1,0,1	2.5% (7/280)	1.7% (6/344)	
Medina 0,1,1	3.9% (11/280)	3.8% (13/344)	
Bifurcation angulation < 70°	13.9% (39/280)	10.5% (36/344)	0.186
Aorto-ostial lesion	3.9% (11/280)	3.2% (11/344)	0.622
Severe tortuosity^a^	7.1% (20/280)	0% (0/344)	< 0.001
Lesion length > 20 mm^b^	37.3% (91/244)	24.0% (72/300)	< 0.001
Heavy calcification^c^	22.5% (63/280)	23.5% (81/344)	0.758
Thrombus	0% (0/280)	0% (0/344)	NA
SYNTAX score 2020^d^			
Predicted MACE for 5 years (%)			
PCI	23.6 ± 13.6	23.8 ± 12.0	0.771
CABG	16.2 ± 9.4	15.4 ± 8.4	0.040
Absolute risk difference	7.4 ± 6.8	8.4 ± 5.9	0.192
Predicted mortality for 5 years (%)			
PCI	15.7 ± 12.6	15.4 ± 11.0	0.595
CABG	11.0 ± 8.8	10.5 ± 8.0	0.048
Absolute risk difference	4.7 ± 4.9	4.9 ± 4.1	0.597
Predicted mortality for 10 years (%)			
PCI	31.3 ± 20.1	31.4 ± 18.5	0.953
CABG	23.3 ± 16.0	22.4 ± 15.0	0.042
Absolute risk difference	8.0 ± 6.4	8.9 ± 6.0	0.186

*CABG* coronary artery bypass graft, *CCTA* coronary computed tomography angiography, *MACE* major adverse cardiac events, *PCI* percutaneous coronary intervention

^a^ ≥ 1 bend of ≥ 90 degrees, or ≥ 3 bends of 45–90 degrees proximal to the diseased segment.

^b^Total occlusions were excluded in the overall assessment of the length of the lesions

^c^For computed tomography angiography defined as the presence of calcium that encompasses more than 50% of the cross-sectional area of the vessel at any location within the specific lesion. For invasive coronary angiography defined as multiple persisting opacifications of the coronary wall visible in more than one projection surrounding the complete lumen of the coronary artery at the site of the lesion [17]

^d^Absolute risk difference is calculated by subtracting predicted CABG mortality from PCI mortality

### Predicted mortality according to SS-2020

**Fig. 2:**
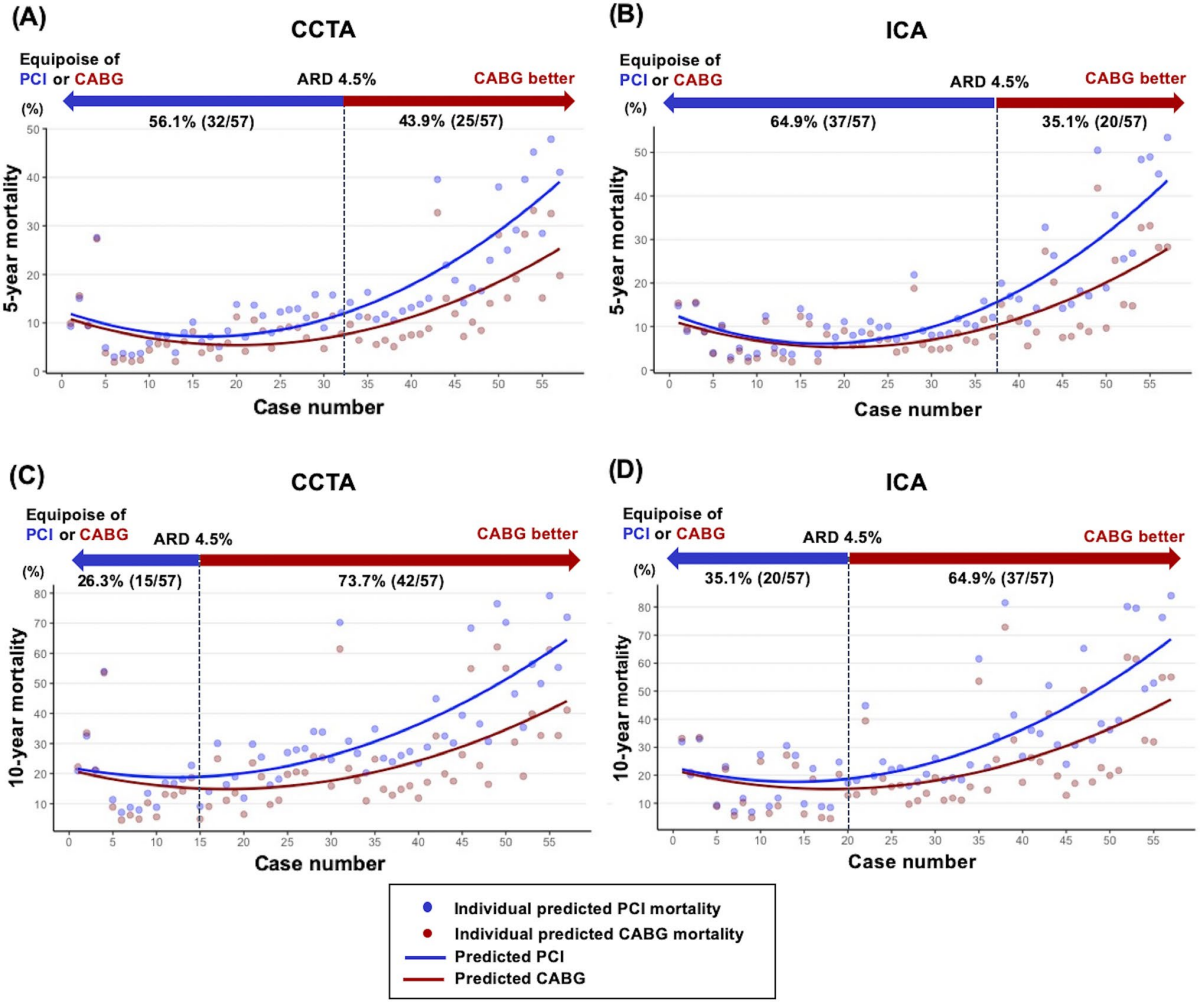
Treatment recommendation according to the individual predicted absolute risk difference. Predicted mortality after either PCI (blue dots) or CABG (red dots) for each patient (individual scatterplots). CABG is recommended for 43.9% of the population on CCTA (**A**), and 35.1% on ICA (**B**) for 5-year mortality. Similarly, CABG is recommended for 73.7% of the population on CCTA (**C**), and 64.9% on ICA (**D**) for 10-year mortality. *ARD* absolute risk difference, *CABG* coronary artery bypass graft, *CCTA* coronary computed tomography angiography, *ICA* invasive coronary angiography, *PCI* percutaneous coronary intervention

The predicted mortality with PCI or CABG according to the SS-2020, as well as the predicted individual ARD in all-cause mortality at 5- and 10-year, ranked in order of magnitude based on CCTA and ICA, is shown in a scatter plot indicating 5-year (Fig. 2A, B) and 10-year mortality (Fig. 2C, D). Patients on the left side have a low ARD, and hence these patients have equipoise between PCI and CABG. In contrast, those on the right side have a large ARD, such that the treatment benefit of CABG is higher than PCI, and hence CABG is recommended for these patients. Using the ARD cut-off of 4.5% results in CABG being the recommended treatment to lower 5-year mortality in 43.9% of patients when using CCTA (Fig. 2A) versus 35.1% when using ICA (Fig. 2B). Similarly, for 10-year mortality CABG is safer than PCI for 73.7% of the population on CCTA (Fig. 2C) compared to 64.9% on ICA (Fig. 2D). Bland–Altman analysis for the predicted mortality according to SS-2020 derived from ICA and CCTA are shown in Supplementary Fig. 2.

### Agreement in treatment recommendation between predicted absolute risk difference derived from ICA and CCTA

**Fig. 3:**
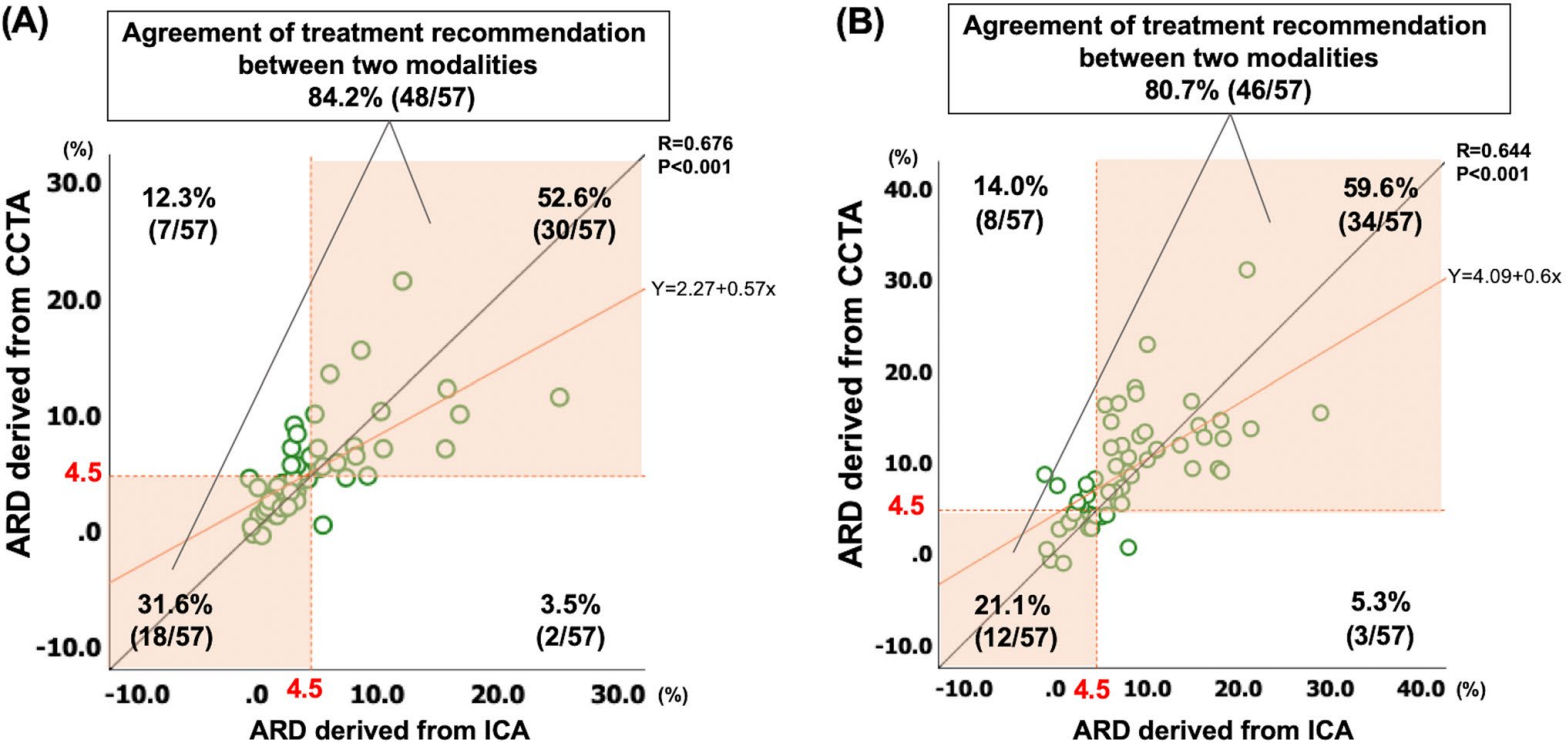
Scatter plots of individual absolute risk difference between two modalities. Scatter plots between the two modalities for 5-year (**A**) and 10-year all-cause mortality (**B**) according to the personalized SYNTAX score 2020. The orange line shows the regression line, and the orange dotted line shows absolute risk difference of 4.5%. Orange areas show diagnostic agreement between the two modalities. The agreements of treatment recommendations among the two modalities are 84.2% for 5-year (**A**), and 80.7% for 10-year all-cause mortality (**B**). *ARD* absolute risk difference, *CCTA* coronary computed tomography angiography, *ICA* invasive coronary angiography

**Fig. 4:**
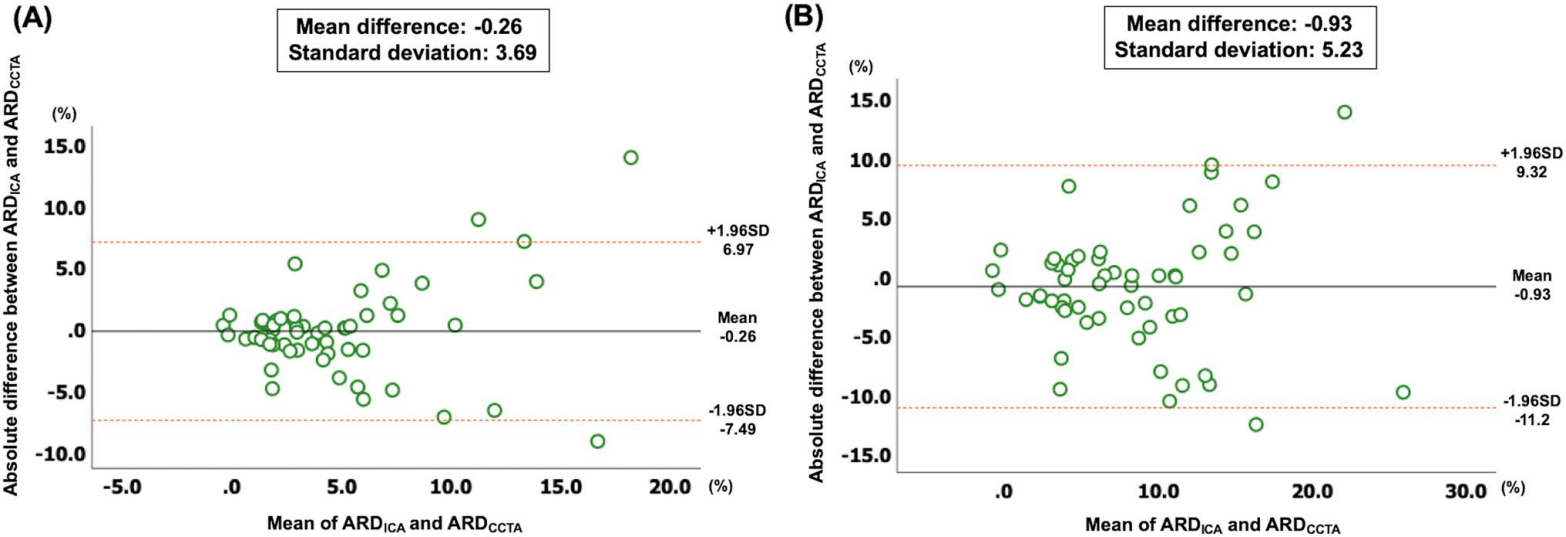
Agreement of absolute risk difference between two modalities. Bland–Altman analysis between absolute risk difference derived from invasive coronary angiography and coronary computed tomography angiography for 5-year (**A**), and 10-year all-cause mortality (**B**). Black line shows the mean difference, and the orange dotted lines show 95% CI. *ARD* absolute risk difference, *CCTA* coronary computed tomography angiography, *ICA* invasive coronary angiography

The correlation of ARD for predicting 5- and 10-year all-cause mortalities between ICA and CCTA are shown in Fig. 3. According to the individual ARD cut-off of 4.5%, agreement in the recommended treatment was observed in 48 (84.2%) and 46 (80.7%) patients for 5- and 10-year all-cause mortality, respectively. The Pearson correlation coefficient between the ARD derived from CCTA and ICA demonstrated high positive correlations for predicting 5- and 10-year all-cause mortality. Bland–Altman analyses (anatomic or 2020) between the ARD derived from ICA and CCTA demonstrated a mean difference of − 0.26 and standard deviation of 3.69 for 5-year mortality, and a mean difference of − 0.93 and standard deviation of 5.23 for 10-year mortality (Fig. 4). The scatter plot and Bland–Altman analysis for 5-year risk of MACE is shown in Supplementary Fig. 3 and Supplementary Fig. 4.

The agreements in revascularization recommendations according to individual ARDs between PCI and CABG are summarized in Table 3. Substantial and moderate agreements were confirmed with Cohens’ kappa of 0.672 (95% CI 0.574–0.770) and 0.551 (95% CI 0.433–0.668) for 5- and 10-year all-cause mortality, respectively. The scatter plots for 5- and 10-year predicted mortalities derived from the two imaging modalities are shown in Supplementary Fig. 5.

**Table 3:** Agreement on treatment recommendation between coronary computed tomography angiography and invasive coronary angiography

	Treatment recommendation based on CCTA	
	CABG	Equipoise CABG and PCI
Treatment recommendation based on ICA		
(A)		
CABG	31.6% (18/57)	3.5% (2/57)
Equipoise CABG and PCI	12.3% (7/57)	52.6% (30/57)
(B)		
CABG	59.6% (34/57)	5.3% (3/57)
Equipoise CABG and PCI	14.0% (8/57)	21.1% (12/57)

Treatment recommendation for predicting 5-year (A) and 10-year mortality (B). (A) Cohen's Kappa 0.672 (95% CI 0.574–0.770). The agreement of revascularization treatment: 84.2%. (B) Cohen's Kappa 0.551 (95% CI 0.434–0.668). The agreement of revascularization treatment: 80.7%

*CABG* coronary artery bypass graft, *CCTA* coronary computed tomography angiography, *ICA* invasive coronary angiography, *PCI* percutaneous coronary intervention

## Discussion

The main findings from the present study, which to the best of our knowledge is the first report assessing the diagnostic performance of SS-2020 based on CCTA in patients with 3VD with or without LMCAD are:

1. The anatomical SYNTAX scores derived from ICA and CCTA were comparable.
2. There were moderate to substantial agreements between revascularization decisions made using the SS-2020 derived using CCTA (Cohen’s Kappa 0.672 for 5-year mortality and 0.551 for 10-year mortality) and conventional ICA.
3. Although, a significant difference was identified in predicted CABG mortality, the Bland–Altman analysis demonstrated acceptable agreement between the two modalities with no significant differences in individual ARDs as predicted by the SS-2020.

Several studies have compared the anatomical SYNTAX score derived from CCTA and ICA and reaffirmed that its calculation using CCTA is reasonably accurate and reproducible, especially in non-complex patients [5, 18–20], however some discordance between the two has been seen in heavy calcified plaque and the Medina classification of bifurcation lesions [21–23]. The present study also showed comparable total anatomical SYNTAX scores between the two modalities, as the tendency for CCTA to overestimate the severity of stenosis—due to the blooming effect of calcium—and the resulting increased number of lesions was tempered by ICA identifying more lesions > 20 mm, more trifurcation lesions (due to the lack of the optimal angiographic view) and more severe tortuosity (due to the two-dimensional perception of the tortuosity).

In the future CCTA may play a pivotal role as a “one-stop-shop” in screening, diagnosis, decision making, and treatment planning [24]. Recently the Discharge (Diagnostic Imaging Strategies for Patients with Stable Chest Pain and Intermediate Risk of Coronary Artery Disease) trial provided the first randomized comparison between invasive and non-invasive diagnostic assessment among patients with stable chest pain and an intermediate pre-test probability of CAD [25]. At medium-term follow-up, whilst the risk of MACE was diverging it was only non-significantly lower with CCTA compared to ICA, however the frequency of major procedure-related complications was lower with an initial CCTA strategy. The SCOT-HEART trial showed that the use of CCTA in patients presenting with stable chest pain resulted in a lower subsequent risk of death from CAD or non-fatal myocardial infarction than standard care alone. The SYNTAX III REVOLUTION trial demonstrated that in patients with LMCAD or 3VD virtually treatment decisions based on CCTA using the SYNTAX score II were in almost perfect agreement (Cohen’s Kappa 0.82; concordance 92%) with those derived from ICA [6], and this was the foundation of the ongoing FASTRACK CABG trial, that assesses the real feasibility and safety of surgical planning and execution based solely on CCTA findings. Given that the guidelines have shifted to a ‘CT first strategy’, it is time to evaluate how clinicians can select and execute the best revascularization treatment by utilizing CCTA [1]. The selection by clinicians of a preferred therapy, when two treatment options are available, relies predominantly on an “average treatment benefit,” traditionally provided by an ARD (or treatment benefit) derived from Kaplan–Meier analysis in randomized trials. However, the goal remains to identify who in a heterogeneous population, subjected to novel diagnostic modalities and treatment decisions, will benefit or be harmed by this new approach and who will have an equipoise outcome [7].

In the present study, we used the SS-2020 derived from CCTA to predict the best treatment considering personalized risk and treatment benefit and compared that selection with the one derived from ICA. Since the anatomical SYNTAX score (effect modifier) and the presence of LMCAD/3VD type of disease (effect modifier) were comparable between the two imaging modalities, the ARDs according to the SYNTAX 2020 were not significantly different, resulting in agreements of treatment recommendations for 5- and 10-year mortality of 84.2% and 80.7%, respectively. These results indicate that CCTA could be used as an alternative to ICA to make decisions regarding the modality of revascularization and to predict MACE and all-cause mortality. We acknowledge that 15.8% and 19.3% of the proportions had discordance in treatment recommendation between the two modalities of imaging. In the probabilistic formula predicting mortality based on the SS-2020 (Appendix), the difference in predicted mortality was partially determined by the two so-called “effect modifiers”—the presence of LMCAD/3VD and anatomical SYNTAX score when comparing the two imaging modalities in the same patient. In the present study, anatomical SYNTAX score and the occurrence of LMCAD/ 3VD were not statistically significant between the two modalities; however, there was a tendency for LMCAD to be more frequently diagnosed in ICA than CCTA, (5.4% vs 2.5%, P = 0.074). This difference in imaging modalities might be implicated in the discordance in predicted mortality and thereby in treatment decision-making. In the original SYNTAX III REVOLUTION trial, the agreement in treatment recommendation based on the SYNTAX score II between the two modalities reached 93%, which was higher than our current results [6] and can be explained by the difference in calibration and discrimination between the two prediction models. The SS-2020 showed better discrimination and calibration for outcomes and the treatment benefit of CABG over PCI compared with the original SYNTAX score II, implying that it could provide better treatment recommendations compared to the SYNTAX score II [7].

There were several limitations to this study. First, clinical outcomes were not available. Only probabilistic prediction was calculated; therefore, we could not assess the calibration and discrimination of the two modalities versus MACE or mortality. Secondly, several subgroups were under-represented, such as female or patients with reduced LVEF. Thirdly, although external validation of the SS-2020 in the most contemporary cohort of the CREDO-Kyoto registry has shown the persisting actuality and accuracy of the probabilistic model [13], clinicians should realize that additional risk factors, such as biomarkers, physical and mental states, active malignancy, frailty, and severe other co-morbid conditions that are strong predictors of mortality, are not accounted for in the SS-2020 [26]. Surgical ineligibility in itself is an independent predictor of increased mortality even after adjustment for important surgical risk scores such as the EuroSCORE or the Society of Thoracic Surgeons (STS) score, indicating that these surgical risk scores may be insufficient for determining surgical ineligibility [26–28]. Finally, in the FASTTRACK CABG trial, only one type of CT scanner (256-slice GE Healthcare REVOLUTION CT) was used, and this may raise the potential issue of generalizability of our results to clinical practices with less multi-slice CT expertise and usage of lower quality CT scanners.

## Conclusion

In patients with 3VD, with or without LMCAD, there was moderate to substantial agreement between treatment recommendations based on the SS-2020 derived using CCTA and ICA, suggesting that CCTA could be used as an alternative to ICA when making decisions regarding the modality of revascularization.

### Supplementary Information

Below is the link to the electronic supplementary material.

Supplementary file1 (DOCX 17409 kb)

## Abbreviations

ARD: Absolute risk difference
CABG: Coronary artery bypass graft
CAD: Coronary artery disease
CCTA: Coronary computed tomography angiography
ICA: Invasive coronary angiography
LMCAD: Left main coronary artery disease
MACE: Major adverse cardiovascular events
PCI: Percutaneous coronary intervention
SYNTAX: Synergy between percutaneous coronary intervention with taxus and cardiac surgery

## References

[1] Knuuti J, Wijns W, Saraste A (2020). 2019 ESC Guidelines for the diagnosis and management of chronic coronary syndromes. Eur Heart J.

[2] Writing Committee M, Gulati M, Levy PD (2021). 2021 AHA/ACC/ASE/CHEST/SAEM/SCCT/SCMR guideline for the evaluation and diagnosis of chest pain: a report of the American College of Cardiology/American Heart Association Joint Committee on Clinical Practice Guidelines. J Am Coll Cardiol.

[3] Serruys PW, Morice MC, Kappetein AP (2009). Percutaneous coronary intervention versus coronary-artery bypass grafting for severe coronary artery disease. N Engl J Med.

[4] Zorlak A, Zorlak A, Thomassen A (2015). Patients with suspected coronary artery disease referred for examinations in the era of coronary computed tomography angiography. Am J Cardiol.

[5] Papadopoulou SL, Girasis C, Dharampal A (2013). CT-SYNTAX score: a feasibility and reproducibility study. JACC Cardiovasc Imaging.

[6] Collet C, Onuma Y, Andreini D (2018). Coronary computed tomography angiography for heart team decision-making in multivessel coronary artery disease. Eur Heart J.

[7] Takahashi K, Serruys PW, Fuster V (2020). Redevelopment and validation of the SYNTAX score II to individualise decision making between percutaneous and surgical revascularisation in patients with complex coronary artery disease: secondary analysis of the multicentre randomised controlled SYNTAXES trial with external cohort validation. Lancet.

[8] Kawashima H, Pompilio G, Andreini D (2020). Safety and feasibility evaluation of planning and execution of surgical revascularisation solely based on coronary CTA and FFRCT in patients with complex coronary artery disease: study protocol of the FASTTRACK CABG study. BMJ Open.

[9] Andreini D, Pontone G, Mushtaq S (2018). Image quality and radiation dose of coronary CT angiography performed with whole-heart coverage CT scanner with intra-cycle motion correction algorithm in patients with atrial fibrillation. Eur Radiol.

[10] Andreini D, Takahashi K, Mushtaq S (2022). Impact of coronary calcification assessed by coronary CT angiography on treatment decision in patients with three-vessel CAD: insights from SYNTAX III trial. Interact Cardiovasc Thorac Surg.

[11] Serruys PW, Onuma Y, Garg S (2009). Assessment of the SYNTAX score in the syntax study. EuroIntervention.

[12] Takahashi K, van Klaveren D, Steyerberg EW, Onuma Y, Serruys PW (2021). Concerns with the new SYNTAX score – authors' reply. Lancet.

[13] Hara HSH, van Klaveren D, Kent DM (2021). External validation of the SYNTAX Score II 2020. J Am Coll Cardiol.

[14] McHugh ML (2012). Interrater reliability: the kappa statistic. Biochem Med (Zagreb).

[15] Fleiss JL (1973). The equivalence of weighted Kappa and the intraclasscorrelation coefficient as measures of reliability. Educ Psycol Meas.

[16] Bland JM, Altman DG (1995). Comparing methods of measurement: why plotting difference against standard method is misleading. Lancet.

[17] Sianos G, Morel MA, Kappetein AP (2005). The SYNTAX Score: an angiographic tool grading the complexity of coronary artery disease. EuroIntervention.

[18] Ugur M, Uluganyan M, Cicek G (2015). The reliability of computed tomography-derived SYNTAX score measurement. Angiology.

[19] Suh YJ, Hong YJ, Lee HJ (2015). Accuracy of CT for selecting candidates for coronary artery bypass graft surgery: combination with the SYNTAX Score. Radiology.

[20] Collet C, Onuma Y, Miyazaki Y, Morel MA, Serruys PW (2017). Integration of non-invasive functional assessments with anatomical risk stratification in complex coronary artery disease: the non-invasive functional SYNTAX score. Cardiovasc Diagn Ther.

[21] Grodecki K, Opolski MP, Staruch AD (2020). Comparison of computed tomography angiography versus invasive angiography to assess medina classification in coronary bifurcations. Am J Cardiol.

[22] Cavalcante R, Onuma Y, Sotomi Y (2017). Non-invasive Heart Team assessment of multivessel coronary disease with coronary computed tomography angiography based on SYNTAX score II treatment recommendations: design and rationale of the randomised SYNTAX III Revolution trial. EuroIntervention.

[23] Collet C, Miyazaki Y, Ryan N (2018). Fractional flow reserve derived from computed tomographic angiography in patients with multivessel CAD. J Am Coll Cardiol.

[24] Serruys PW, Hara H, Garg S (2021). Coronary computed tomographic angiography for complete assessment of coronary artery disease: JACC state-of-the-art review. J Am Coll Cardiol.

[25] Maurovich-Horvat P, Bosserdt M, Group DT (2022). CT or invasive coronary angiography in stable chest pain. N Engl J Med.

[26] McNulty EJ, Ng W, Spertus JA (2011). Surgical candidacy and selection biases in nonemergent left main stenting: implications for observational studies. JACC Cardiovasc Interv.

[27] Michel P, Roques F, Nashef SA, EuroSCORE Project Group (2003). Logistic or additive EuroSCORE for high-risk patients?. Eur J Cardiothorac Surg.

[28] Shahian DM, O'Brien SM, Filardo G (2009). The Society of Thoracic Surgeons 2008 cardiac surgery risk models: part 1–coronary artery bypass grafting surgery. Ann Thorac Surg.

