# Supplementary material for: Treatment recommendation based on SYNTAX score 2020 derived from coronary computed tomography angiography and invasive coronary angiography
Source: Int J Cardiovasc Imaging. 2023 Jun 27;39(9):1795–804. doi: 10.1007/s10554-023-02884-0 (PMC10519866; doi:10.1007/s10554-023-02884-0)
Supplement: Supplementary file 1 — Supplementary file1 (DOCX 17409 kb) [file 10554_2023_2884_MOESM1_ESM.docx]

**Supplementary materials**

**Supplementary Figure 1**

**Flow-chart of the FASTTRACK CABG trial.**


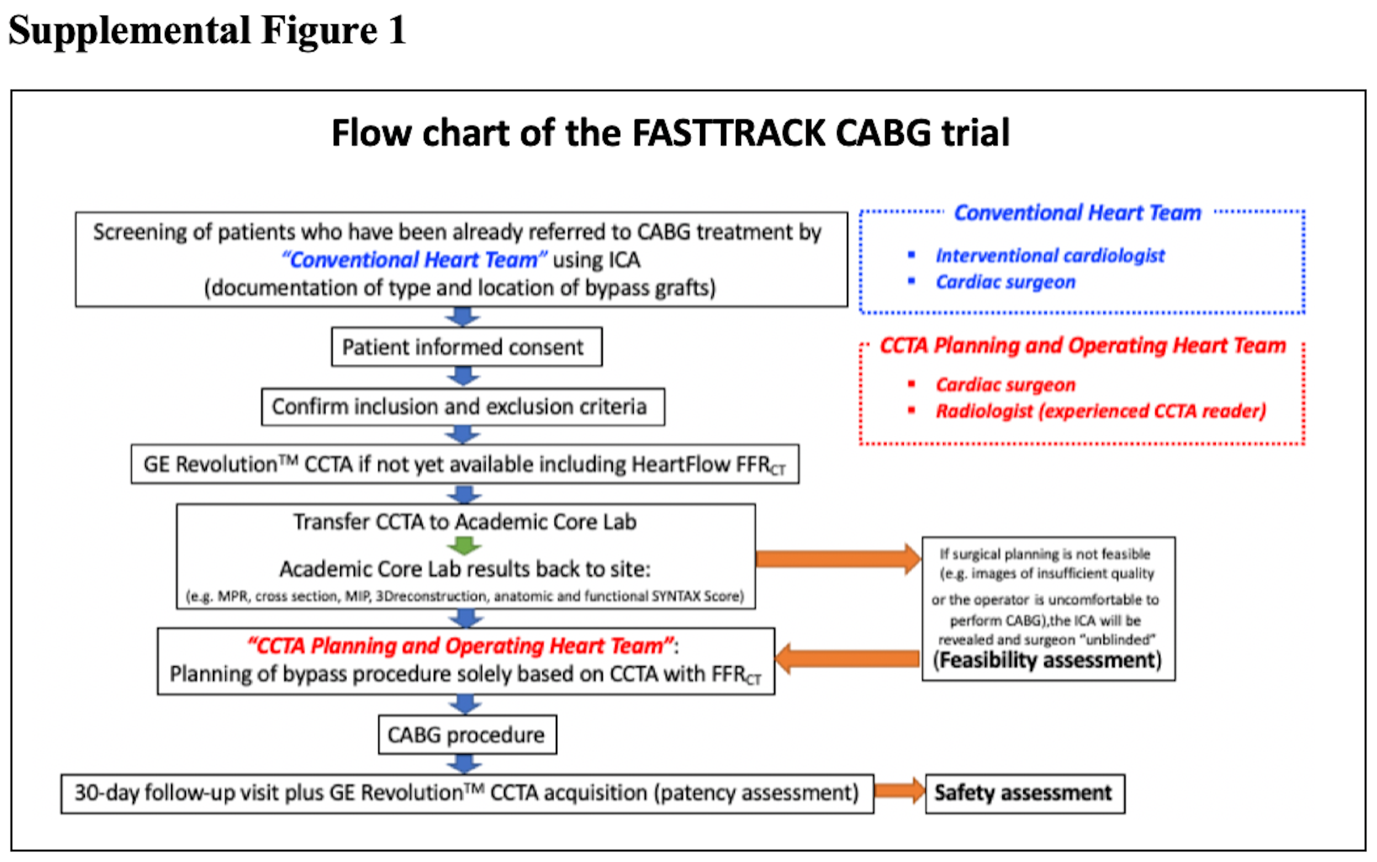


Abbreviations:

CABG: coronary artery bypass graft, CCTA: coronary computed tomography angiography, FFR_CT_: fractional flow reserve computed tomography, ICA: invasive coronary angiography,

**Supplementary Figure 2**

**Bland-Altman analysis for the predicted mortality between the two modalities.**

Five-year predicted PCI (A) and CABG (B) mortality. Ten-year predicted PCI (C) and CABG (D) mortality.


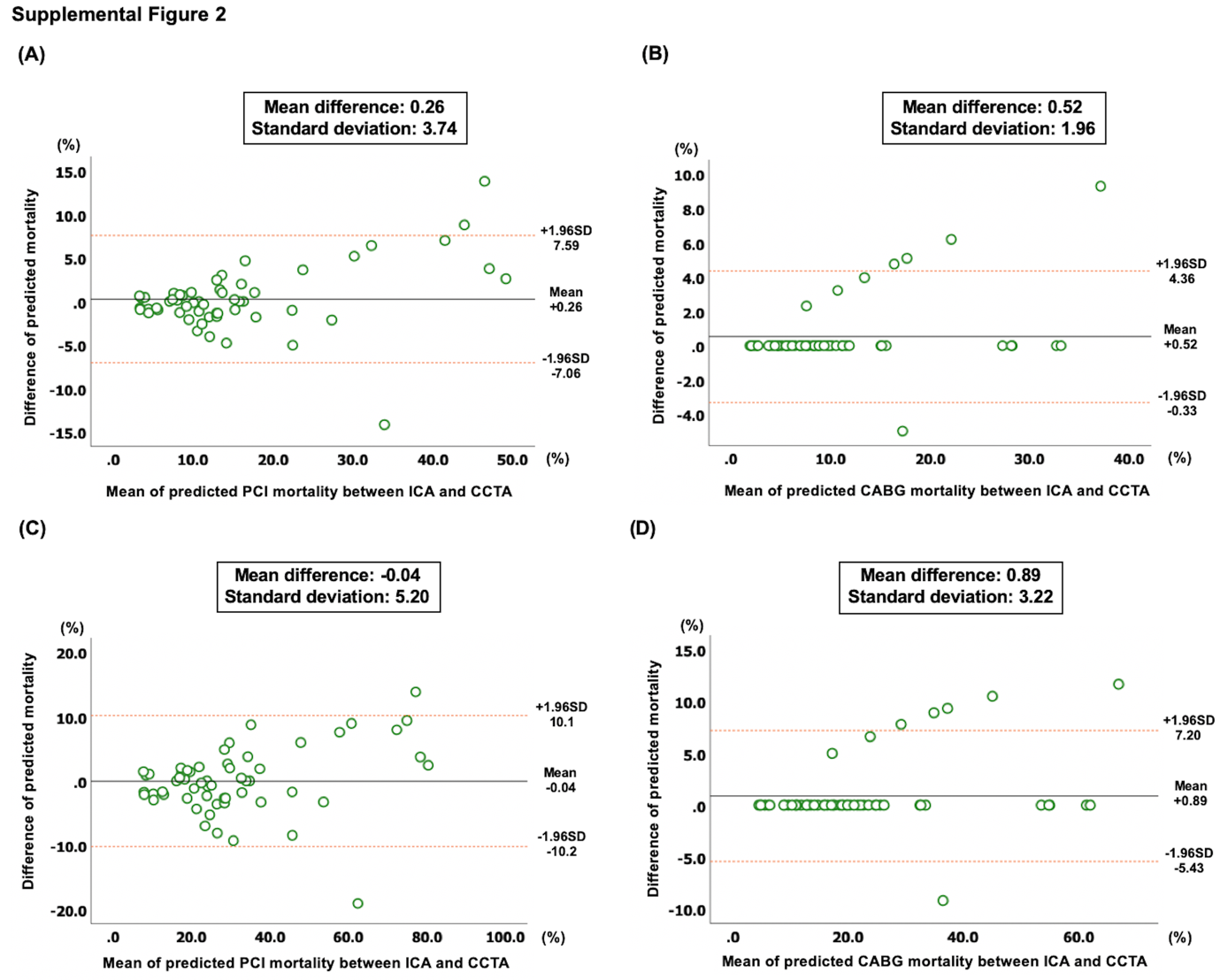


Abbreviations:

CABG: coronary artery bypass graft, CCTA: coronary computed tomography angiography, ICA: invasive coronary angiography PCI: percutaneous coronary intervention

**Supplementary Figure 3**

**Scatter plot of individual absolute risk difference between the two modalities.**

Scatter plot and correlation of absolute risk differences derived from invasive coronary angiography and coronary computed tomography angiography for 5-year having a risk of major adverse cardiac events according to the SYNTAX score 2020. The Spearman correlation coefficient is 0.594.

**
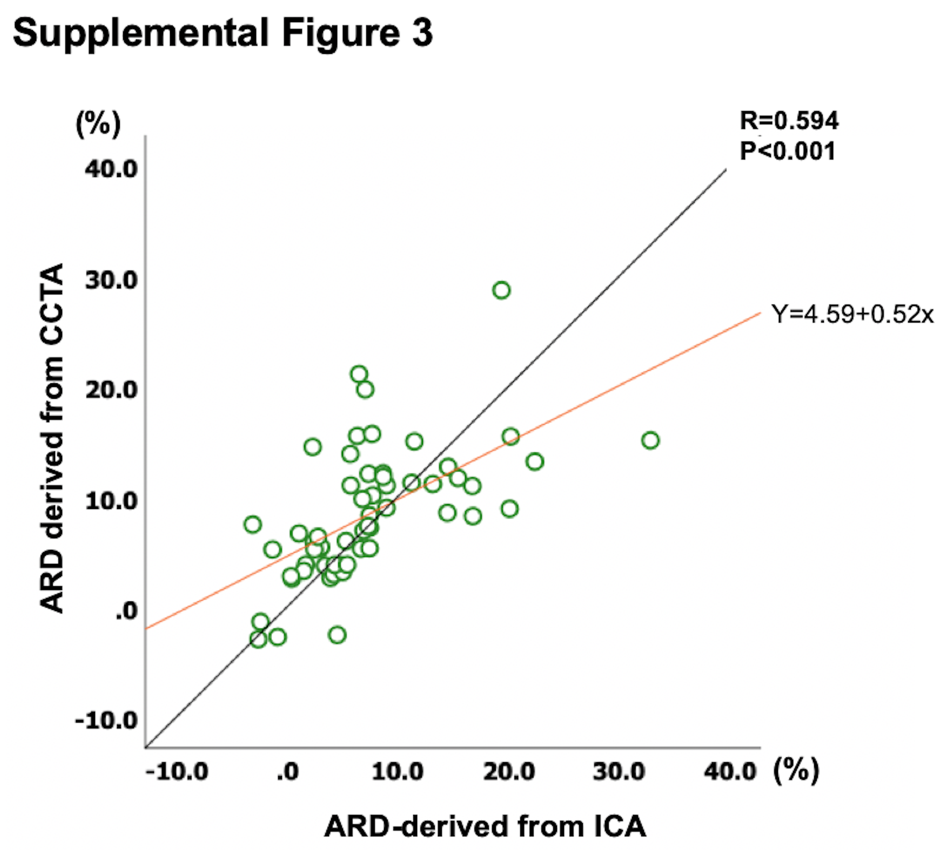
**

Abbreviations:

ARD: absolute risk difference, CCTA: coronary computed tomography angiography, ICA: invasive coronary angiography

**Supplementary Figure 4.**

**Agreement of absolute risk difference between the two modalities.**


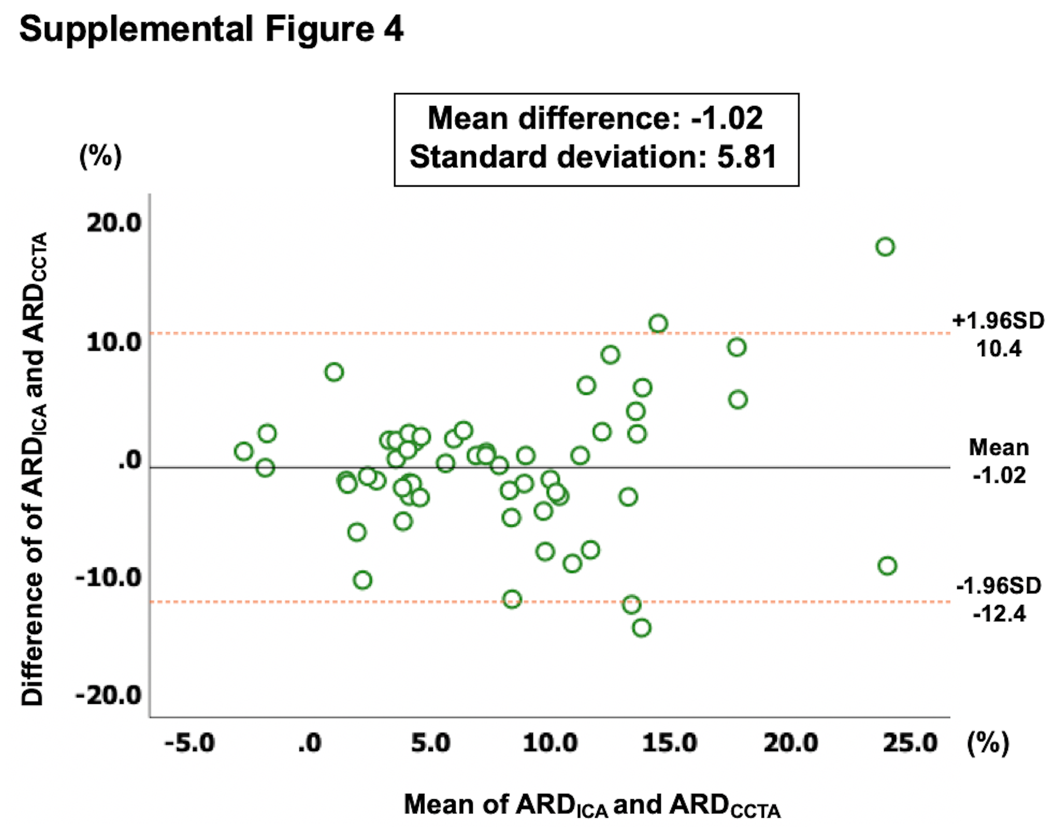
Bland-Altman analysis between absolute risk difference derived from invasive coronary angiography and coronary computed tomography angiography for 5-year having a risk of major adverse cardiac events according to the SYNTAX score 2020. Black line shows the mean difference, and the orange dotted lines show 95% CI.

Abbreviations:

ARD: absolute risk difference, CCTA: coronary computed tomography angiography, ICA: invasive coronary angiography

**Supplementary Figure 5**

**Scatter plot of predicted mortality between the two modalities.**

Scatter plot and correlation of predicted mortality derived from invasive coronary angiography and coronary computed tomography angiography for 5- and 10-year mortalities according to the SYNTAX score 2020.

Five-year predicted CABG (A) and PCI (B) mortalities. Ten-year predicted CABG (C) and PCI (D) mortalities.


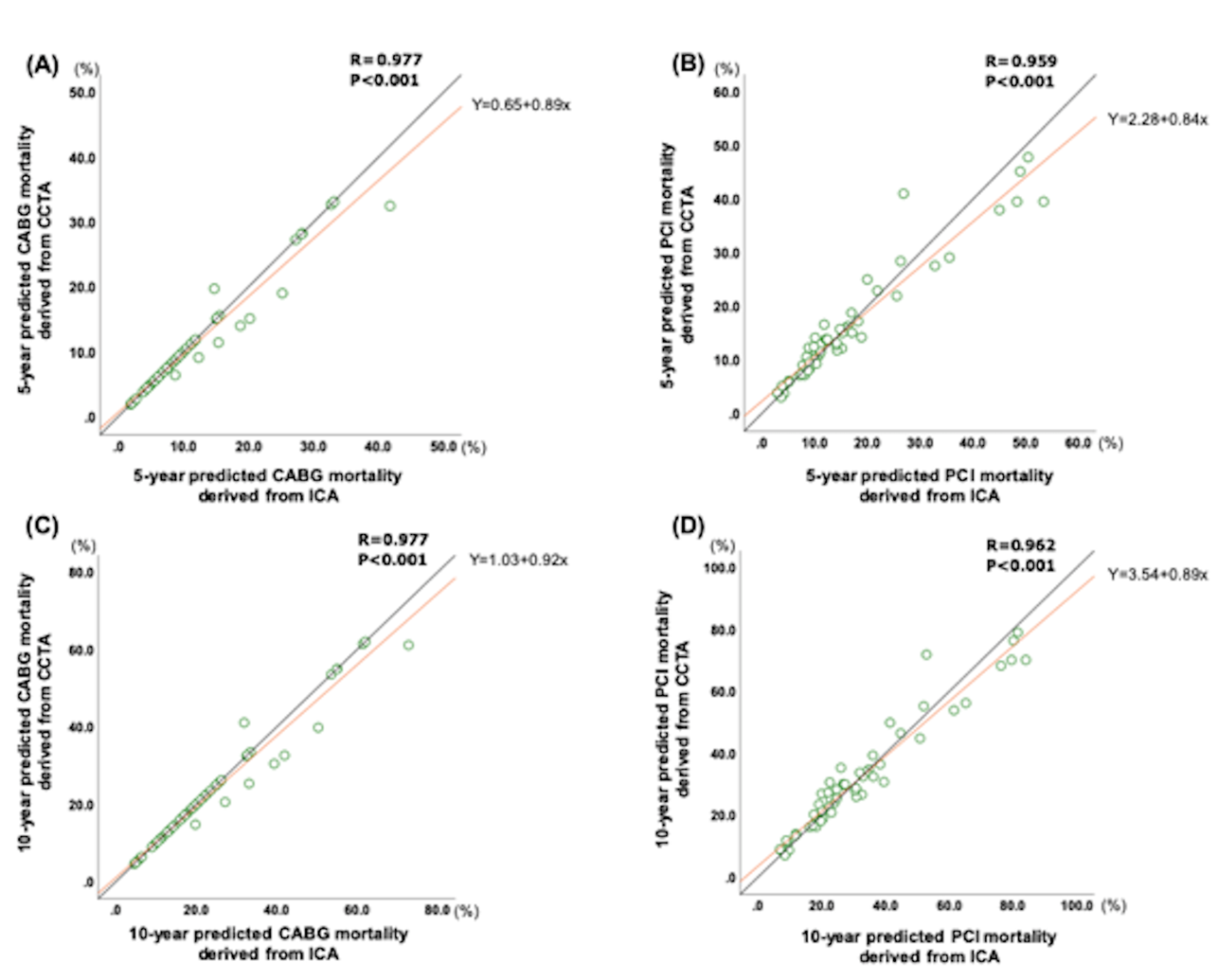


Abbreviations:

CABG: coronary artery bypass grafts, CCTA: coronary computed tomography angiography, ICA: invasive coronary angiography, PCA: percutaneous coronary intervention, SYNTAX: Synergy Between PCI with TAXUS and Cardiac Surgery

**Appendix**

Models of SYNTAX score 2020 for predicting risk of all-cause death at 5 and 10 years for percutaneous coronary intervention and coronary artery bypass grafting.

***Predicted probability of all-cause death at 5 years for PCI***

=1 – exp(–0·101 × exp[0·99 × {0·72 × age / 10 – 0·07 × creatinine clearance / 10 – 0·31 × left ventricular ejection fraction / 10 + 0·48 × chronic obstructive pulmonary disease + 0·73 × peripheral vascular disease + 0·20 × medically treated diabetes + 0·46 × on insulin + 0·66 × current smoker} – 0·1 × LMCAD + 0·16 × {SYNTAX score – 29}/10 – 2.8])

***Predicted probability of all-cause death at 5 years for CABG***

=1 – exp(–0·101 × exp[0·99 × {0·72 × age / 10– 0·07 × creatinine clearance / 10– 0·31 × left ventricular ejection fraction / 10 + 0·48 × chronic obstructive pulmonary disease + 0·73 × peripheral vascular disease + 0·20 × medically treated diabetes + 0·46 × on insulin + 0·66 × current smoker} – 0·4 × 3VD – 0·08 × LMCAD – 2.8])

***Predicted probability of all-cause death at 10 years for PCI***

=1 – exp(–0.243 × exp[0.99 × {0.72 × age / 10 – 0.07 × creatinine clearance / 10 – 0.31 × left　ventricular ejection fraction / 10 + 0.48 × chronic obstructive pulmonary disease + 0.73 × peripheral vascular disease + 0.20 × medically treated diabetes + 0.46 × on insulin + 0.66 × current smoker} – 0.1 × LMCAD + 0.16 × {SYNTAX score–29}/10 – 2.8])

***Predicted probability of all-cause death at 10 years for CABG***

=1 – exp(–0.243 × exp[0.99 × {0.72 × age / 10 – 0.07 × creatinine clearance / 10 – 0.31 × left　ventricular ejection fraction / 10 + 0.48 × chronic obstructive pulmonary disease + 0.73 × peripheral vascular disease + 0.20 × medically treated diabetes + 0.46 × on insulin + 0.66 × current smoker} – 0.4 × 3VD – 0.08 × LMCAD – 2.8])

Abbreviations:

LMCAD: left main coronary artery disease, SYNTAX: Synergy Between PCI with TAXUS and Cardiac Surgery, VD: vessel disease
